# A Hybrid Textile Electrode for Electrocardiogram (ECG) Measurement and Motion Tracking

**DOI:** 10.3390/ma11101887

**Published:** 2018-10-02

**Authors:** Xiang An, George K. Stylios

**Affiliations:** Research Institute for Flexible Materials, Heriot-Watt University, Edinburgh TD1 3HF, UK; G.Stylios@hw.ac.uk

**Keywords:** textile electrode, ECG, motion sensor, skin-electrode impedance

## Abstract

Wearable sensors have great potential uses in personal health monitoring systems, in which textile-based electrodes are particularly useful because they are comfortable to wear and are skin and environmentally friendly. In this paper, a hybrid textile electrode for electrocardiogram (ECG) measurement and motion tracking was introduced. The hybrid textile electrode consists of two parts: A textile electrode for ECG monitoring, and a motion sensor for patient activity tracking. In designing the textile electrodes, their performance in ECG measurement was investigated. Two main influencing factors on the skin-electrode impedance of the electrodes were found: Textile material properties, and electrode sizes. The optimum textile electrode was silver plated, made of a high stitch density weft knitted conductive fabric and its size was 20 mm × 40 mm. A flexible motion sensor circuit was designed and integrated within the textile electrode. Systematic measurements were performed, and results have shown that the hybrid textile electrode is capable of recording ECG and motion signals synchronously, and is suitable for ambulatory ECG measurement and motion tracking applications.

## 1. Introduction

With the miniaturization of electronics, improvements in performance of low-power microprocessors, and the development of artificial intelligence, personal health monitoring systems are becoming possible. Wearable electronics, wireless communications, textile sensors, mobile computing, and cloud computing are becoming increasingly important in personal health monitoring systems. Wearable sensors and textile electrodes are particularly suitable for some long-term health monitoring applications, such as electrocardiogram (ECG) measurement and motion tracking.

Textile electrodes are usually made of conductive yarns by weaving, knitting or embroidering processes; or by coating or printing conductive polymers on non-conductive fabrics. In the studies of textile electrodes, most textile electrodes are knitted structure [1,2,3]. Priniotakis et al. [4] compared the knitted and woven textile electrodes by using an electrochemical cell; the results show that the knitted structure has the lowest contact resistance. Woven and embroidered textile electrodes have also been researched with some success [5]. However, there is no consistent conclusion as to which type of textile structure (knit, woven, embroidered) performs best in ECG recording, because it involves many factors, such as the structure of the fibers and yarns, the fabric density, and the manufacturing process. The conductive material type for making the textile electrode is another important factor that affects the performance of the electrode. Many studies have used silver plated textile materials to make textile electrodes [2,5,6]. Other conductive materials have also been studied [7,8,9,10]. Rattfalt [11] made textile electrodes with 100% stainless steel and 20% stainless steel, which showed the acceptable stability of electrode potentials. However, stainless steel is highly direct current voltage (DC) polarizable and very alloy dependent [12]. Jang et al. [13] explored the possibilities of copper (Cu) sputtered fabric as ECG electrode. Conductive polymers have also been used for making textile electrodes. Pani et al. [14] made textile electrodes with poly(3,4-ethylene dioxythiophene):poly(styrene sulfonate) (PEDOT:PSS) coated woven fabric to monitor ECG signals.

Compared with conventional silver/silver chloride (Ag/AgCl) rigid metal electrodes, textile electrodes have the advantage of being soft, flexible and breathable, allowing the wearer to feel more comfortable than conventional metal plate electrodes in long-term monitoring. In addition, as textile electrodes can be easily integrated into a garment by weaving, knitting or sewing, there is no need for any adhesive to attach on the body, so they are skin friendly (no skin irritation or discomfort) and environmentally friendly (electrodes are reusable). Based on these advantages, many researchers have used textile electrodes in the development of wearable ECG systems [7,15,16,17,18].

In this paper, a hybrid textile electrode is proposed. It consists of two parts: A textile electrode for ECG measurement, and a motion sensor for patient activity tracking. Although there are some studies that combine motion sensors and textile electrodes into a wearable system [17,18,19,20], this is the first time that the motion sensor is directly integrated into the textile electrode. There are good reasons for designing this hybrid textile electrode. First of all, motion signals that recorded in synchrony with ECG signals are beneficial in the diagnosis of heart disease. Some studies [21,22,23,24] have found that heavy physical exertion can be the trigger of the onset of arrhythmia and acute myocardial infarction. Furthermore, changes in posture (sitting up or standing up) may also be the cause of arrhythmia, known as postural tachycardia syndrome (PoTS) [25]. Therefore, the motion signals recorded in synchronization with the ECG signals can help the cardiologist find the cause of the heart disease by providing information about the patient’s physical activity when the ECG shows an abnormality. Moreover, tracking daily physical activity and ECG can also help prevent the sudden death in patients with coronary heart disease, because some studies have shown that sudden death is related to physical exertion [26,27,28]. Secondly, since the hybrid textile electrode is placed on the patient’s chest to measure the ECG, the motion sensor on the hybrid electrode can also obtain information about the patient’s respiration by tracking the movement of the chest while the patient remains stationary (sitting or standing). The measured respiration along with the ECG can also be used to diagnosis a common heart disease—respiratory sinus arrhythmia.

Due to the absence of conductive gel/paste, textile electrodes usually have much higher and more unstable skin-electrode impedance than conventional Ag/AgCl wet electrodes. And the complexity and instability of fabric structure itself also make the characteristics of textile electrodes different from conventional metal plate electrodes. Therefore, in this paper, the electrical properties of the dry textile electrode at the skin-electrode interface were first studied. Based on that, the electrode material and size were investigated, and an optimum textile electrode was made. In order to integrate a motion sensor with a textile electrode, a small flexible printed circuit board (FPCB) was designed. A hybrid textile electrode was finally fabricated by integrating an optimum textile electrode with the small flexible motion sensor circuit board.

## 2. The Skin-Electrode Interface of Textile Electrode

**Figure 1:**
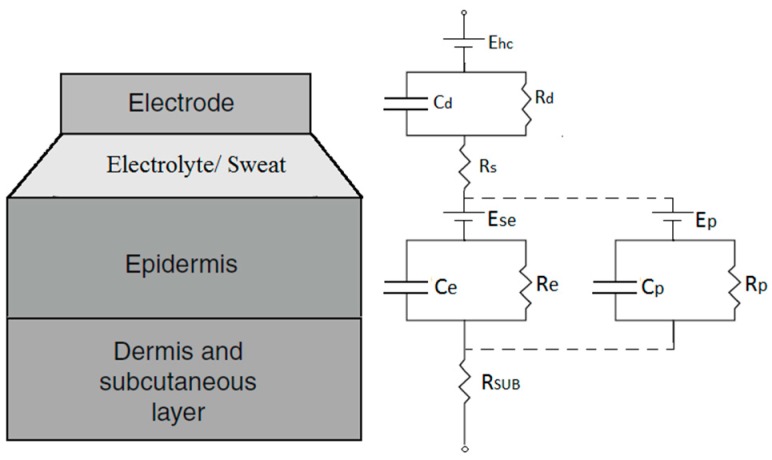
The electrical equivalent circuit of the skin-electrode interface [29].

Textile electrodes, like conventional metallic plate electrodes, are in contact with human skin as electrical conductors. The difference from metallic plate electrodes is that the conductive metal is electroplated onto the textile substance or blended into the yarn. Therefore, the electrochemical reactions occurring at the interface between the conventional metal electrode and the skin also occur at the interface between the textile electrode and the skin. The interface is called the skin-electrode interface. Neuman [29] proposed an equivalent circuit for modelling the electrical characteristics of the skin-electrode interface for conventional metal electrodes, as shown in Figure 1.

In the case of dry textile electrodes, although they do not have a conductive gel/paste on the electrode surface, the skin moisture and perspiration can also be considered as a thin electrolyte layer between the textile electrode and the skin. “Dry” electrodes are really only dry when first applied, skin moisture and perspiration will quickly accumulate under the electrode [30]. Therefore, the equivalent circuit for conventional metal electrodes is also applicable to textile electrodes. According to the equivalent circuit, the skin-electrode impedance of the textile electrode $Z_{\mathrm{Textile}}$ can be calculated as follows:

(1) $$\;Z_{\mathrm{Textile}}=R_{s}+\frac{R_{d}}{1+{j\omega R}_{d}C_{d}}+R_{\mathrm{sub}}+\frac{R_{e}}{1+{j\omega R}_{e}C_{e}},\;$$

where $R_{d}$ represents the charge transfer resistance and $C_{d}$ represents the capacitance across the electrode-electrolyte interface, $R_{e}$ represents the resistance of epidermis layer, $C_{e}$ represents the capacitance induced by the nonconductive stratum corneum layer, $R_{s}$ represents the resistance of the sweat, $R_{\mathrm{sub}}$ represents the overall resistance of the tissue underneath the epidermis layer.

The skin-electrode impedance of the dry textile electrode is usually much higher than the conventional wet electrode. In most cases, the value of $Z_{\mathrm{Textile}}$ is up to several hundred kΩ. Due to the fact that the human skin has a highly nonhomogeneous multi-layered structure, the electrical properties of the skin vary along different body parts, which also mean that the skin-electrode impedances of the two electrodes at different skin locations are generally different. Webster [29] has found that the impedance imbalance introduces noise into ECG signals. Olsen [31] has found that the impedance imbalance was typically 50 percent of the individual skin-electrode impedance. Therefore, the most effective way to reduce the impedance imbalance of dry textile electrodes is to reduce the skin-electrode impedance. Thus, the optimum textile electrode should be made of a material having low skin-electrode impedance characteristics.

## 3. Electrode Material

**Figure 2:**
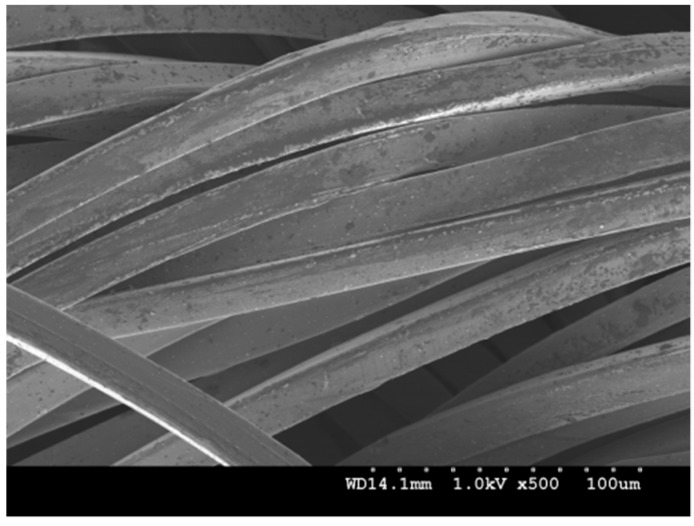
The scanning electron microscope (SEM) micrograph of a silver plated nylon yarn.

Various materials have been used to produce conductive textiles that are either embedded into fabrics as conductive yarns, or plated with electrically conductive components, such as carbon, copper, nickel, or silver. However, when choosing materials that will come into contact with the human skin, as in the case of ECG electrodes, their biocompatibility becomes very important as the electrode is directly applied onto the human body. Different from most other materials, silver is not only innocuous to human skin, but also antibacterial [32,33,34,35]. Therefore, conductive fabrics made from silver plated nylon yarns are favored for making textile electrodes by weaving or knitting. When compared with woven fabrics, knitted fabrics usually are more flexible, stretchable, and can take up easily the curvature of the body when attached. So, in this paper, four different knitted conductive fabrics made from silver plated nylon yarn were considered as electrode materials, shown in Table 1, and their electrical properties were investigated on a skin dummy. Electrode material TE1 is a silver plated knitted fabric purchased from Shieldex (MedTex P-130, Shieldex, Bremen, Germany), material TE2 is made of 4 ply silver plated nylon yarn (235/34 dtex 4-ply, Shieldex, Bremen, Germany), material TE3 is made of 2 ply silver plated nylon yarn (117/17 dtex 2-ply, Shieldex, Bremen, Germany) and material TE4 is a silver plated spacer fabric purchased from Shieldex (Spacer Fabric B, Shieldex, Bremen, Germany). Figure 2 shows the scanning electron microscope (SEM) micrograph of a silver plate nylon yarn. The average diameter of a silver plated nylon monofilament is about 0.028 mm.

### 3.1. Experimental Method

**Figure 3:**
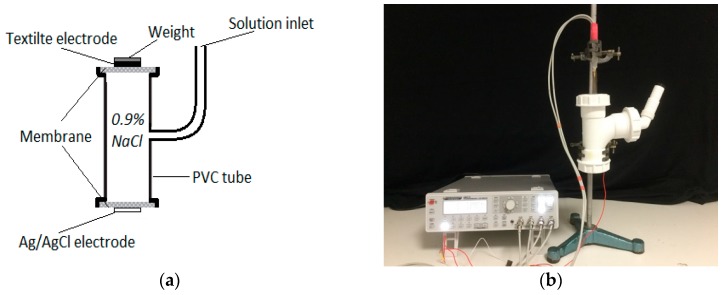
Skin-electrode impedance measurement on a skin dummy: (**a**) Skin dummy; (**b**) test setup.

The electrical properties of human skin have great variations and dependent upon time and location of the skin [36]. Thus, a skin dummy (Figure 3a) is used to measure the skin-electrode impedance to avoid the unwanted impedance variation induced by the human skin. The design of the skin dummy is based on Westbroek’s electrochemical cell [4,37,38], which consists of a Polyvinyl chloride (PVC) tube filled with 0.9% of NaCl solution to simulate the body fluid. Two polyvinylidene fluoride (PVDF) membranes are installed on the two open ends of the PVC tube to simulate the skin barrier between the body fluid and the textile electrodes. The PVDF membranes were obtained from Merck^®^ (Darmstadt, Germany), and are the same membranes that were used in P.J. Xu’s [39] dynamic evaluation system. The pore size of the PVDF membranes in our evaluation system is 100 nm, as this size is large enough to allow the electrolyte to flow freely through the perforated membrane [40].

**Figure 4:**
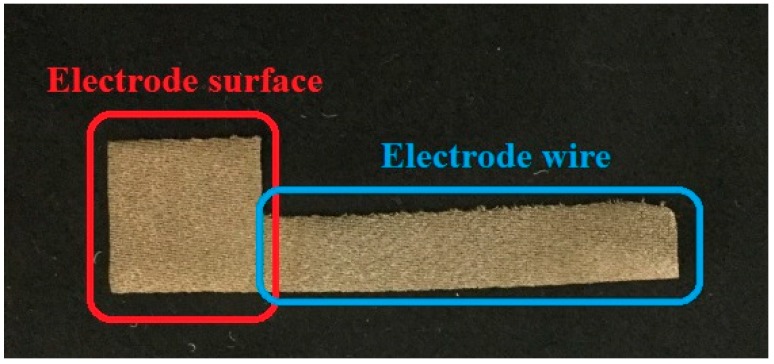
Structure of a textile electrode for the skin-electrode measurement.

Four textile electrodes made from materials TE1, TE2, TE3 and TE4 were tested on the skin dummy, as shown in Table 1. The structure of the textile electrodes used in the measurement, shown in Figure 4, it consists of two parts: A square area of size 15 mm × 15 mm, which is the electrode surface that is in contact with the skin dummy; a rectangular area of size 7 mm × 50 mm is the electrode wire that is connected to an impedance meter. In the measurement, the textile electrode is placed on the top surface of the skin dummy and is fixed with a weight of 100 g, which applied a force of 0.98 N to the electrode. A self-adhesive Ag/AgCl electrode is placed on the bottom surface to serve as a reference electrode. A high-precision LCR-Bridge meter (HM8118, HAMEG instruments, Mainhausen, Germany) is used in this system to measure the impedance. Two measurements were done in here. First, impedances were measured at the frequency of 100 Hz, and measurements were last for one hour. Second, impedances were measured within a frequency range of 20 Hz to 20 kHz when the skin-electrode impedance is stabilized. Measurements were performed in a conditioned laboratory where the room temperature was controlled at 20 ± 2 °C and the relative humidity at 65 ± 2%.

### 3.2. Results and Discussion

**Figure 5:**
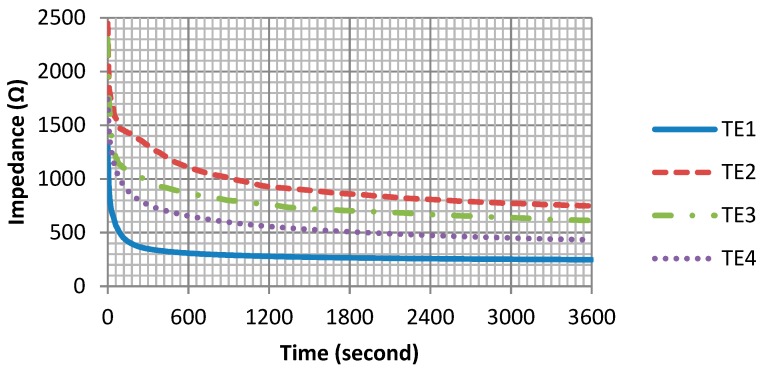
Skin-electrode impedance in 1 h.

As shown in Figure 5, the skin-electrode impedances of all four textile electrodes show a similar trend: Impedances are rapidly decreasing within the first few minutes and then gradually become stable. However, their differences are also noticeable. In the first few minutes of measurement, the electrode TE1 has the fastest impedance drop among the four electrodes, and its impedance tends to be stable in the shortest time. Moreover, after the stabilization period, the skin-electrode impedances of the four electrodes are different, from the highest to the lowest impedance TE2, TE3, TE4, TE1 respectively.

**Figure 6:**
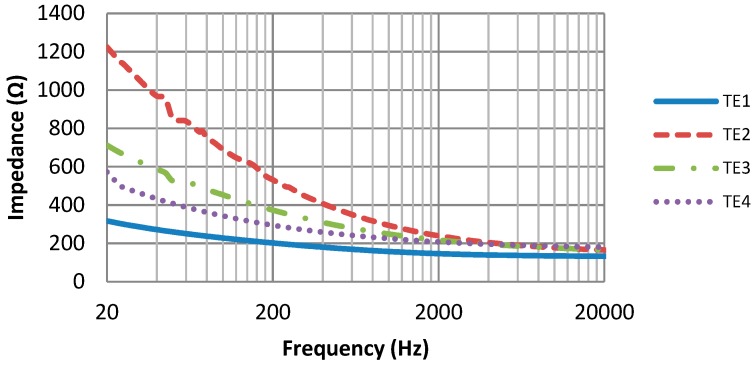
Skin-electrode impedance versus frequency.

Figure 6 shows the skin-electrode impedance over the frequency range. The impedance of the four electrodes is frequency-dependent: As the frequency increases, the impedance decreases, which is consistent with the capacitive behavior of the skin-electrode interface. However, the differences in these four electrode materials are also clearly shown in the impedance curves. Electrode TE1 has the smallest impedance, as well as the smoothest impedance-frequency curve.

**Figure 7:**
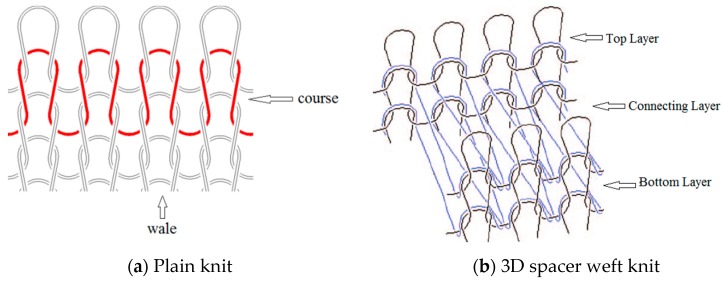
Knitted fabric structure.

The difference in skin-electrode impedance can be explained by the stitch density of these fabrics. As demonstrated in Table 1, the four electrode materials are all made of silver plated yarn, and they are all made by knitting. Textile Electrodes TE1, TE2, TE3 are all weft knitted structures, electrode TE4 is a knitted 3D spacer structure, but its surface layer is also weft knitted, as shown in Figure 7. The most significant difference between these four fabrics is their stitch density and the yarn diameter. Electrode TE1 has the highest stitch density and the smallest yarn diameter, whilst electrode TE2 has the lowest stitch density and the largest yarn diameter. The measured skin-electrode impedance is positively related to the yarn diameter and negatively related to stitch density.

**Figure 8:**
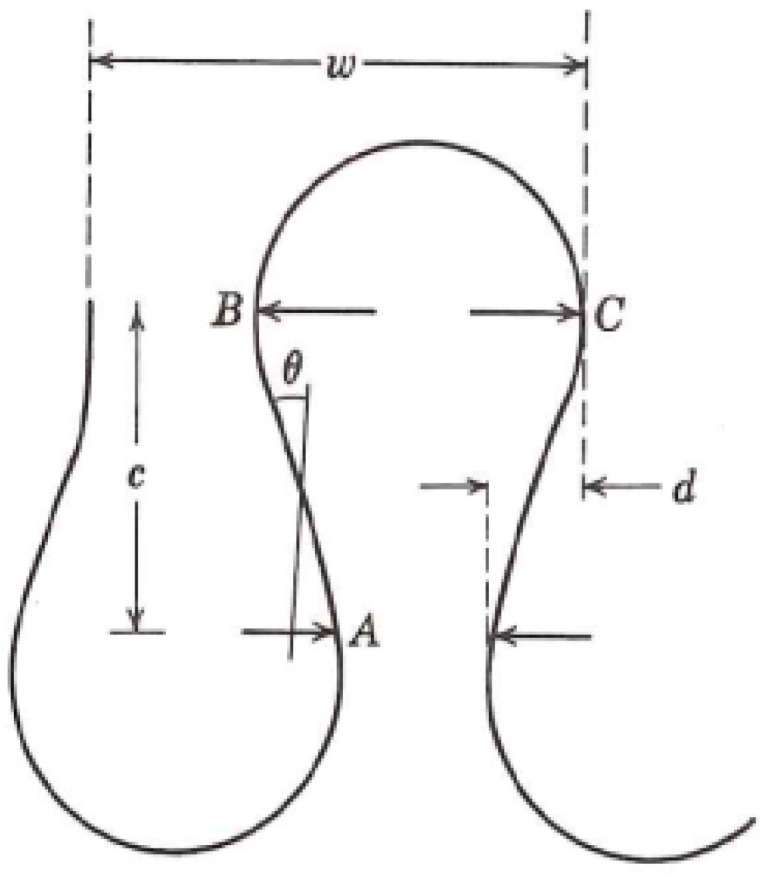
Loop model according to Munden [41].

According to the geometrical model of a plain knitted fabric, suggested by Munden [41], the basic structure of a knitted fabric is a loop that consists of parts of circles joined by straight lines, as shown in Figure 8. This model is based on Peirce’s assumptions [42]: The bending resistance of the yarns was negligible and that the yarn was circular in cross-section.

The length of a single loop can be calculated by Equation (2):

(2) $$\;l=2\cdot l_{AB}+2\cdot l_{BC}\;$$

where $l_{AB}$ is the straight length between point A and point B on the loop, $l_{BC}$ is the semicircle length between point B and point C, *c* is the course spacing, *d* is the yarn diameter. Equation (3) is the calculation of $l_{AB}$ that based on the Peirce’s model of plain weave [43]. Equation (4) is the calculation of $l_{BC}$ that proposed by Munden [41].

(3) $$\;l_{AB}=c\left[1+\frac{9}{16}{\left(\frac{d}{c}\right)}^{2}\right]\;$$

(4) $$\;l_{BC}=0.544\frac{c^{2}}{d}\;$$

Noting that the loop is a 3D structure, and the section of the loop between B and C is actually covered by a higher loop and only the section between A and B and its mirror can directly get contact with the skin. So, the effective skin contact length in a single loop can be estimated by Equation (5):

(5) $$\;l_{1}=2c\left[1+\frac{9}{16}{\left(\frac{d}{c}\right)}^{2}\right].\;$$

Therefore, the effective contact area per square centimeter can be estimated by Equation (6):

(6) $$\;S=2\cdot C\cdot W\cdot c\left[1+\frac{9}{16}{\left(\frac{d}{c}\right)}^{2}\right]\cdot D,\;$$

where *W* is the number of wales per cm, *C* is the number of courses per cm, *c* is the course spacing equals to 10/C, *D* is the effective contact width of the yarn and skin. *D* is related to the yarn diameter, fiber diameter, and the deformation rate of the yarn and the skin.

According to Equation (6), the stitch density C·W is positively related to the effective skin contact area. When the stitch density increases, it actually increases the effective skin-electrode contact area. As we know from the electrical equivalent circuit of the skin-electrode interface in Figure 1, the skin-electrode interface has both resistive behavior and capacitive behavior. The resistive behavior can be expressed by Equation (7), and the capacitive behavior can be expressed by Equation (8):

(7) $$\;R_{e}=\rho \frac{l}{A},\;$$

(8) $$\;C_{e}=\varepsilon \frac{A}{d},\;$$

where ρ is the electrical resistivity of the material, l is the fabric thickness, *A* is the skin-electrode contact area, ε is the permittivity of the dielectric layer, d is the distance between the electrode and the skin. According to Equations (7) and (8), the increase in the effective skin-electrode contact area will results in a decrease in resistance and an increase in capacitance and according to Equation (1), reduction in resistance and increase in capacitance will eventually lead to a reduction in the skin-electrode impedance. Therefore, increasing the stitch density can effectively reduce the skin-electrode impedance. In addition, the increased stitch density also helps to accumulate sweat under the electrode, so that the skin-electrode impedance drops in a shorter time, meaning a shorter impedance stabilization period.

In ECG monitoring, small skin-electrode impedance means small noise interference; a smooth impedance-frequency curve means that low-frequency signals have less amplitude distortion. So according to our results, electrode material TE1 shows the best performance among all four electrode materials, not only because it has the smallest skin-electrode impedance, but also because it has the smoothest impedance-frequency curve and the shortest impedance stabilization period. Therefore, conductive fabric TE1 is an optimum material for making the hybrid textile electrode. 

## 4. Electrode Size

The size of the electrode has also been reported as having a significant influence on skin-electrode impedance and on the ECG signal’s quality [44]. Puurtinen et al. [45] studied different sizes of textile electrodes and found that the skin-electrode impedance increases with decreasing of the electrode size. Marozas et al. [9] also found that textile electrodes with a contact area smaller than 4 cm^2^ might cause distortions to the signal’s low-frequency spectrum. Therefore, in order to choose the optimum electrode size of the proposed hybrid electrode, the electrode size and its influence on the ECG signal have to be investigated.

### 4.1. Experimental Method

Three different electrode sizes were investigated, the electrodes were all made from conductive fabric TE1. Conventional wet ECG electrodes (2228, 3M, Minnesota, USA) were also used to measure the ECG for comparison purposes. Table 2 listed the areas and dimensions of the four different electrodes.

ECG signals were measured with these four different electrodes on the chest of a female subject. All ECG signals were recorded using ADS1292ECG-FE (Texas Instruments, Dallas, TX, USA), with only the 50 Hz notch filter operating and all other filters switched off. The sample rate of the signal was 500 Hz, a reference electrode was used to reduce the common-mode noise. All electrodes were secured on the skin with a 30 mmHg pressure applied by an elastic chest band.

### 4.2. Results and Discussion

**Figure 9:**
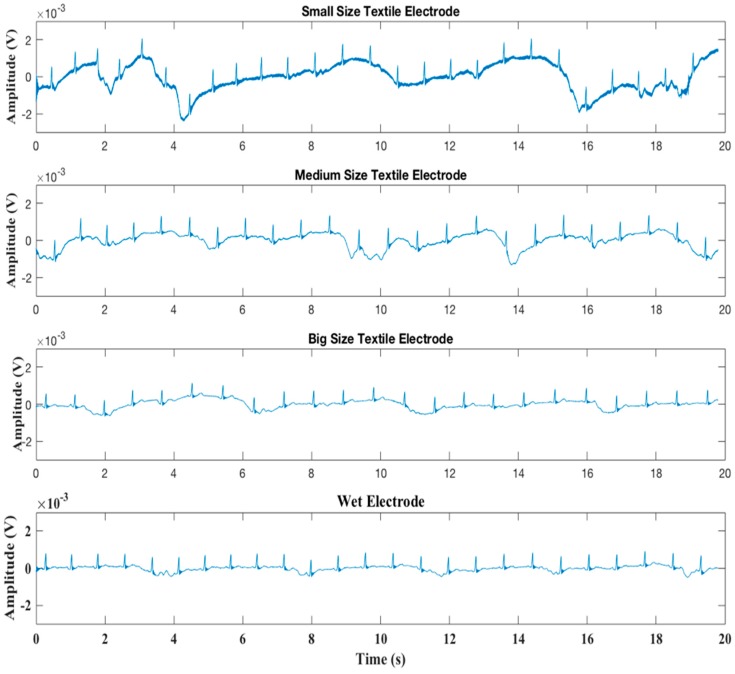
Resting electrocardiogram (ECG) signals recorded by different electrodes.

**Figure 10:**
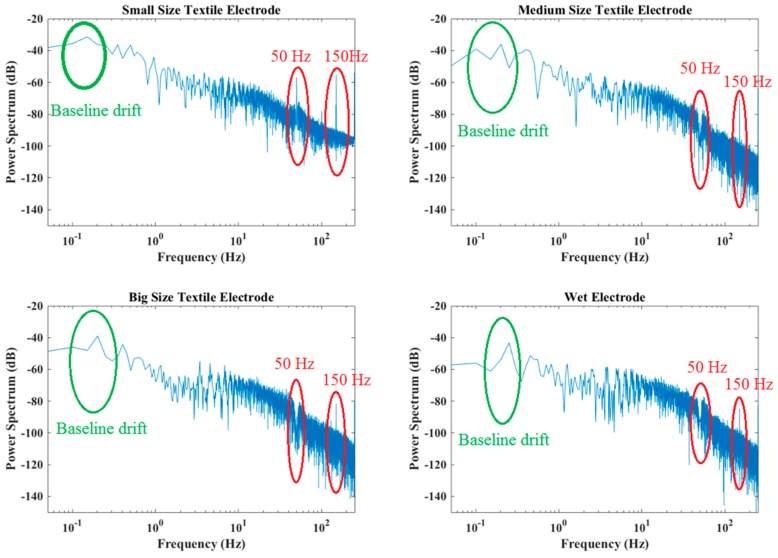
Power spectral density.

Figure 9 shows the original ECG signals recorded with three pairs of dry textile electrodes and one pair of wet electrodes, Figure 10 shows the power spectral density of all ECG signals. As can be seen from Figure 9, baseline drift exists in all ECG signals, this is mainly due to body respiration and its effect on body volume change causing skin-electrode impedance imbalance. As the size of the electrode increases, the baseline drift effect caused by respiration is significantly reduced. Small size textile electrodes have the largest baseline drift compared to other electrodes. Figure 10 shows that the energy of the drifted baseline is in the frequency range of 0–0.5 Hz, and that the smallest textile electrode has the largest baseline drift noise. Besides the baseline drift, high-frequency noise can also be observed in all graphs, because of the presence of electromagnetic fields in the vicinity of the patient. The smallest dry textile electrode introduces more high-frequency noise than all others. However, the high-frequency interference is also decreased with increased electrode size. Although the ECG signal has been filtered by a 50 Hz notch filter, the 50 Hz alternating current (AC) power line interference and its third harmonic (150 Hz) are clearly observed in the spectrum of the smallest size electrode. However, in the spectrum of the largest size electrode, only the 150 Hz harmonic frequency can be observed, and its energy level is similar with that of the conventional wet electrode.

In comparison with wet electrodes, dry textile electrodes usually introduce more noise (including the baseline drift noise and the AC power line interference) into the ECG signals. However, the result for the largest size textile electrode is comparable to that of the wet electrode, meaning that the dry textile electrode having a large electrode size can perform equally well for ECG monitoring. Therefore, the electrode size of 2 cm × 4 cm is an optimum size for making our hybrid textile sensor.

## 5. Motion Sensor FPCB

**Figure 11:**
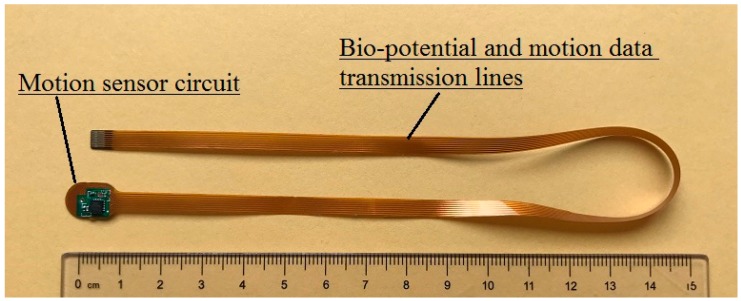
The flexible printed circuit.

**Figure 12:**
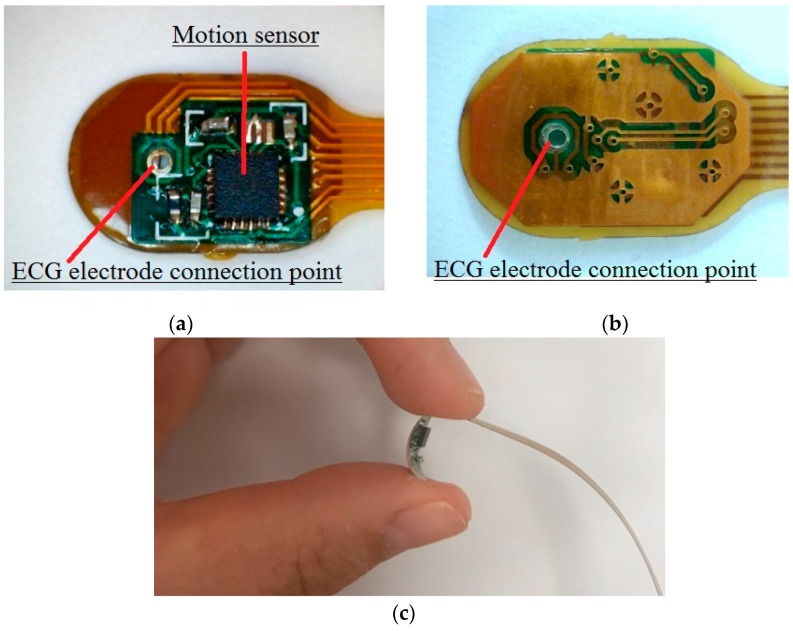
The motion sensor mini flexible printed circuit board (FPCB): (**a**) Top view; (**b**) rear view; (**c**) the flexibility of the FPCB.

To track human activity, microelectromechanical (MEMS) motion sensor MPU-9250 (InvenSense, Calgary, AB, Canada) was used in the design of the hybrid textile electrode, because of its miniature size and powerful features. The MPU-9250 is a multi-chip module consisting of a 3-Axis gyroscope, 3-axis accelerometer, 3-axis magnetometer and a digital motion processor all in a small 3 × 3 × 1 mm package. In order to integrate the motion sensor with the textile electrode, a flexible printed circuit board (FPCB) is specially designed. The FPCB offers power supplies to the MPU-9250 and transmits the detected motion data from the sensor to the microcontroller (MCU). The printed transmission lines in the FPCB also serve as an electrode lead between the textile electrode and the input of the amplifier. As shown in Figure 11, the FPCB consists of two parts: The first part is a mini-circuit board with electronic components on it; the second part is printed transmission lines for transmitting bio-potential signals and motion data to the MCU. The size of the first part is 10 mm × 7 mm. Figure 12: (a) shows the top view of the FPCB; (b) shows the rear view of the FPCB; and (c) shows the flexibility of the FPCB.

## 6. Fabrication of the Hybrid Textile Electrode

**Figure 13:**
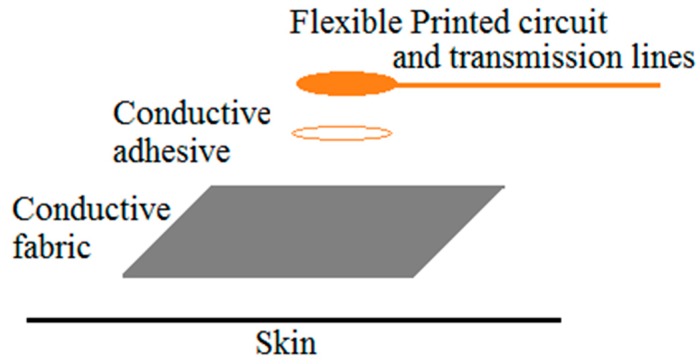
The layout of the hybrid textile electrode.

**Figure 14:**
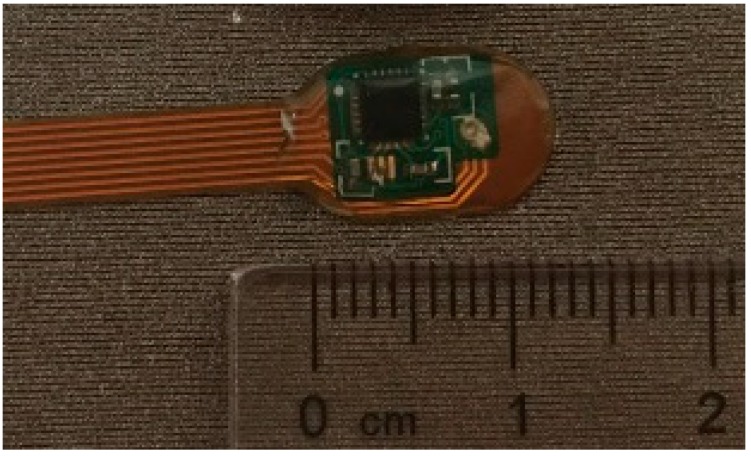
The integration of the circuit board and the conductive fabric.

In order to integrate the mini FPCB into the textile electrode, we used silver conductive adhesive (CW2400, Chemtronics, Kennesaw, GA, USA) to bond the rear side of the mini circuit board with the rear side of the conductive fabric together, to establish a coherent ECG signal transmission line, as demonstrated in Figure 13. The biopotential signal is transmitted from the textile electrode through the silver conductive adhesive layer to the ECG electrode connection point of the FPCB, and it then reaches the input of the biopotential amplifier through the printed transmission line. Electrical insulation is also important for making the hybrid textile electrode. A thick, transparent, flexible silicon coating (FSC, Electrolube, Leicestershire, UK) is brushed on the top surface of the mini circuit board to provide insulation of the electronic components with the outside world. The flexible silicon coating is a solvent based conformal coating designed to protect printed circuit boards. As seen in Figure 14, the FPCB is integrated with the conductive fabric.

**Figure 15:**
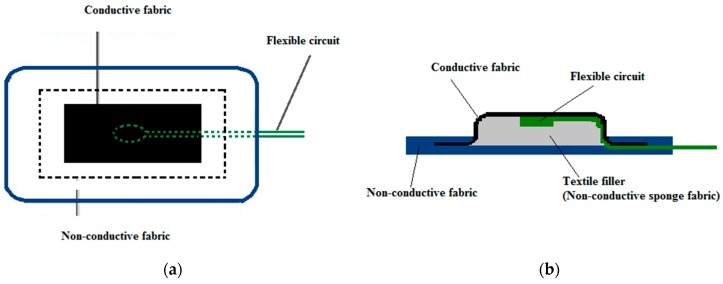
The illustration of the structure of textile electrode: (**a**) Top view; (**b**) side view.

**Figure 16:**
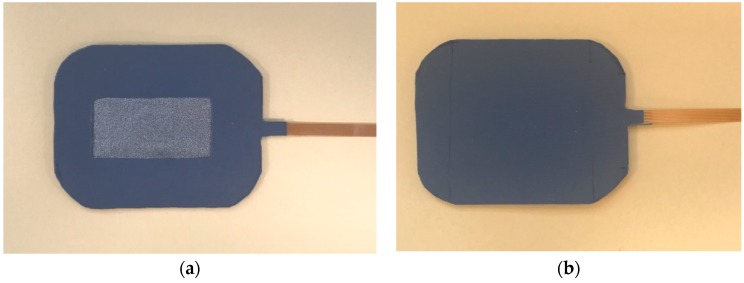
Hybrid textile electrode: (**a**) Top view; (**b**) rear view.

Non-conductive fabric and non-conductive sponge filler fabric are also used for making the hybrid textile electrode. Non-conductive fabric serves as an insulating layer. Non-conductive sponge fabric provides support for the flexible circuit board and the conductive fabric. Figure 15 illustrates the top and the cross-sectional views of the hybrid textile electrode. The middle rectangular area is the conductive fabric surrounded by the non-conductive fabric. The skin contact area of the conductive fabric is 20 mm × 40 mm. Figure 16 shows the top view and the rear view of the hybrid textile electrode.

## 7. Hardware Setup

**Figure 17:**
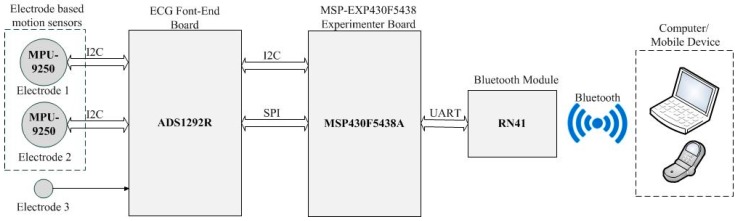
The system setup.

The measurement system configuration is shown in Figure 17. It is based on an MSP 430 microcontroller (Texas Instruments, Dallas, TX, USA) with build in high-performance 12-bit Analog-to-digital converter (ADC) for data acquisition and system control. A low power, 24-bit Analog front-end ADS1292R (Texas Instruments, Dallas, TX, USA) is used for the ECG measurements; and a motion tracking device MPU-9250 (InvenSense, Calgary, AB, Canada) is embedded in the textile electrode to track movement. The measured ECG and motion signals are transferred into a Bluetooth module RN41 (Microchip, Chandler, Arizona, USA) and send to a remote computer or mobile device wirelessly.

## 8. The Implementation of the Hybrid Textile Electrode for ECG Monitoring and Motion Tracking

**Figure 18:**
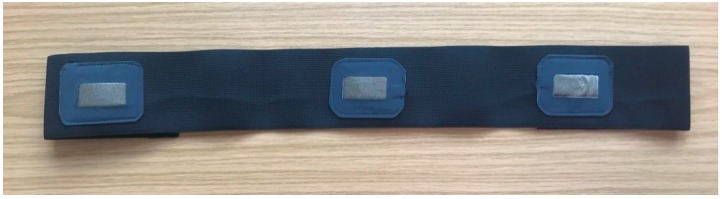
The chest band.

In order to test the performance of the hybrid textile electrodes, we have undertaken measurement using a female subject. Two hybrid textile electrodes and one textile electrode with the same structure were sewn onto an elastic chest band, as seen in Figure 18. The Electrodes were placed on the subject’s chest and were secured onto the skin with a 30 mmHg pressure, applied by an elastic chest band. Signals were recorded under two everyday activities: Sitting and walking.

**Figure 19:**
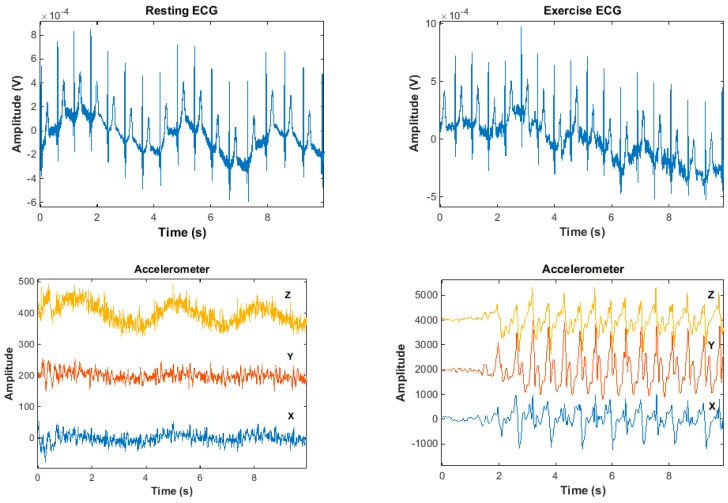

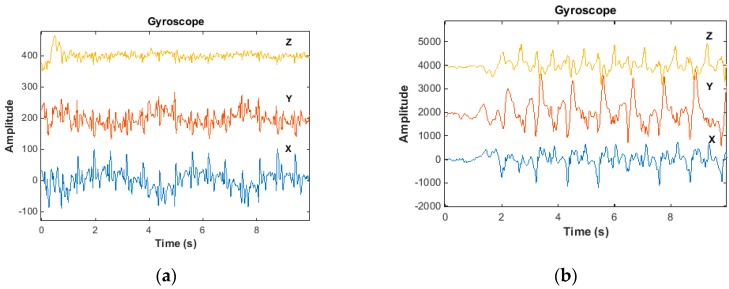
Signals recorded by the hybrid textile electrode: (**a**) Sit; (**b**) walk.

Figure 19 shows the original, unfiltered signal that is recorded with the hybrid textile electrodes. Figure 19a shows the resting ECG and its corresponding motion data; Figure 19b shows the exercise ECG and its corresponding motion data. All ECG signals present baseline drift and high-frequency noise. The baseline drift is mainly caused by the respiration, as the hybrid textile electrodes were integrated into a chest band, the chest movement during respiration induces motion artefacts into the ECG signals. As shown in Figure 19a, chest movement during respiration was captured by the motion sensor in synchronization with the ECG. The accelerometer’s data on the Z axis has the same trend as the baseline drift of the resting ECG. The gyroscope’s data on the X axis also shows a similar trend. The high-frequency noise in ECG signals is mainly induced from power line interference, because textile electrodes have high and unbalanced skin-electrode impedance, thus introducing differential mode noise into the ECG signals. The waveform of exercise ECG shows more interference than the resting ECG, due to motion artefacts caused by walking motion. As shown in Figure 19b, walking motion was captured by the motion sensor in synchronization with the ECG. The accelerometer data and the gyroscope data illustrate the motion pattern.

According to the results presented in Figure 19, the hybrid textile electrode is capable of recording ECG and tracking motion at the same time. Although the recorded ECG signals were contaminated by baseline drift and high-frequency noises, the magnitude of the noise did not corrupt the morphology of the recorded ECG. Therefore, the presence of these noises is tolerable and the performance of the hybrid textile electrode in ECG monitoring is reasonable, and the integrated motion sensor was very accurate in capturing movement by the hybrid textile electrode.

## 9. Conclusions

This paper presents a new hybrid textile electrode that integrates motion sensor MPU9250 with a textile-based electrode. This proposed hybrid textile electrode is not only suitable for long-term ECG monitoring, but also capable of tracking the patient activity simultaneously.

In the design of the hybrid textile, the performances of textile electrodes have been studied. Four electrode materials were investigated, and the conductive fabric TE1 was chosen to be the optimum electrode material. According to the skin-electrode impedance measurement, the conductive fabric TE1 not only has the smallest skin-electrode impedance, but also has the smoothest impedance-frequency curve and the shortest impedance stabilization period. The study on the electrode size has proven that dry textile electrodes with the size of 2 cm × 4 cm can perform equally well as commercial wet electrodes in ECG monitoring. Therefore, the size of the hybrid textile electrode was found optimum at 2 cm × 4 cm. In order to integrate the motion sensor MPU9250 with the textile base, a flexible printed circuit board (FPCB) has been specially designed for this purpose. The size of the FPCB is only 1 cm × 0.7 cm, which makes it easy to integrate into the textile electrode.

The combination of motion signals and ECG signals offers great potential for cardiology clinical trials cardiac rehabilitation patient care, and in general wellbeing and sports. The motion signals recorded in synchronization with the ECG signals can help the cardiologist find the cause of the heart disease by providing information about the patient’s physical activity when the ECG shows an abnormality. By tracking daily physical activity and alerting the patient when the patient is over-exercised can also help prevent the sudden death in patients with coronary heart disease. Furthermore, sports and exercise can also be monitored with these new technologies that can provide better data than the commonplace heart beat monitors.

## Figures and Tables

**Table 1:** The properties of the four selected conductive knitted fabrics.

Electrode Materials		Components	Structure	Fabric Thickness (mm)	Yarn Diameter (mm)	Wales/cm	Courses/cm
TE1	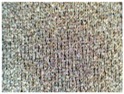	78% silver plated nylon 66 + 22% Elastomer	Weft knitted	0.45 ± 10%	0.13 ± 20%	28/cm	30/cm
TE2	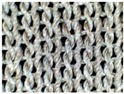	100% silver plated nylon 66	Weft knitted	1.25 ± 10%	0.60 ± 20%	5/cm	6/cm
TE3	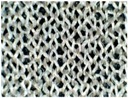	100% silver plated nylon 66	Weft knitted	0.70 ± 10%	0.30 ± 20%	8/cm	12/cm
TE4	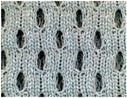	94% silver plated nylon 66 + 6% elastomer	Weft knitted 3D spacer	2.50 ± 10%	0.18 ± 20%	17/cm (surface)	28/cm (surface)

**Table 2:** Electrodes in different size.

	Large Textile Electrode	Medium Textile Electrode	Small Textile Electrode	Conventional Wet Electrode
Shape of Electrodes	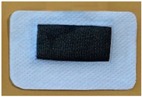	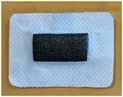	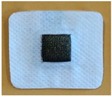	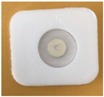
Electrode Area (cm^2^)	8	4.5	2.25	2.27
Electrode Dimension	2 × 4 (w × L, cm)	1.5 × 3.0 (w × L, cm)	1.5 × 1.5 (w × L, cm)	1.7 (φ, cm)
